# Retrospective study of the success of dental implants placed in HIV-positive patients

**DOI:** 10.1186/s40729-019-0174-6

**Published:** 2019-06-13

**Authors:** Nadine Cordero Rubinstein, Zhimon Jacobson, Gail Link McCausland, Serge Dibart

**Affiliations:** 1 University of Maryland School of Dentistry, 650 W. Baltimore Street, Baltimore, MD 21201 USA; 2 Department of Restorative Sciences & Biomaterials, Boston University, GSDM, Boston, USA; 3 Department of Periodontology, Boston University, Boston, USA; 4 Boston University, Boston, MA USA

**Keywords:** HIV-positive patients, Antiretroviral therapy, Bone loss, Dental implant

## Abstract

**Background:**

Data related to HIV-positive patients with dental implants is short-termed and limited. Recent data showed that both HIV and antiretroviral therapy (ART) could lead to low bone mineral density (BMD). The aim of this study was to determine the success rate of dental implants in HIV-positive patients.

**Materials and methods:**

Dental files of 67 HIV-positive patients were selected and reviewed retrospectively, and 18 subjects agreed to return for examination. All implants were evaluated using periapical radiographs that were calibrated to measure bone loss. Crestal bone loss, mobility, and lack of infection were the parameters used to determine implant success.

**Results:**

All dental implants evaluated lacked mobility and infection. Bone loss averaged 0.5 mm in 3.6 years. Subjects were consistent with maintenance and oral hygiene.

**Conclusions:**

Within the limitations of this study, the results suggest that the placement of dental implants on HIV-positive patients is safe and effective.

## Background

HIV infection continues to be a life-threatening disease. In 2009, an estimated 2.6 million newly infected cases were reported [1]. Although the growth rate has plateaued in the last decade, numbers still run high [2]. Prevention efforts, scientific research, and the development of new medication have led to improve the quality and life expectancy of HIV-positive patients [1, 3, 4, 5].

Antiretroviral therapy has been proven to be a life-saving approach for many millions infected [1, 6]. Advances in HIV treatment have improved since the first antiretroviral, zidovudine, in 1987. A monotherapy of nucleoside reverse transcriptase inhibitor (NRTI) provided dramatic survival benefit but did not sustain viral progression. In the 1990s, protease inhibitors (PI) changed the course of HIV epidemic. Combination therapy led to rapid reduction of HIV RNA and improved immune function. In 2014, there are 28 antiretroviral drugs belonging to six different mechanistic classes. Older agents were replaced by new drugs that are more potent, less toxic, and less dosing frequency [1].

Advances in the last and availability of antiretroviral therapy have led to dramatic reductions in the mortality and morbidity of HIV patients [4]. Antiretroviral therapy is also effective in lowering the risk of mother-to-child transmission as well as a post-exposure prophylaxis measure for individuals exposed to HIV [1, 2, 7].

Current knowledge suggests that both, HIV and antiretroviral therapy, are likely to contribute to bone disorders, such as osteopenia and osteoporosis [7, 8, 9]. The virus itself affects osteoblast and osteoclast function. Antiretroviral therapy, especially the initial dosages, seems to accelerate bone mineral loss [10, 11].

Unlike orthopedic implants which are in a closed environment, dental implants have direct communication to the oral cavity. Exposure to microflora of the mouth in conjunction with immunosuppression may affect the long-term outcome of dental implants [1].

Despite the adverse effects, the use of antiretroviral therapy has led HIV-positive patients to maintain low viral loads and normal CD4 counts making them more likely to opt for an elective surgery such as dental implants. Systematic review by Ata-Al et al. [12] mentions the prognosis of dental implants in HIV-positive patients to be similar to that of HIV-negative patients. Strietzel et al. [13] concluded that no modification in the dental routine is required for HIV-positive patients. Oliveira et al. [14], in a pilot study, compared 12-month implant success in 25 HIV-positive patients with different antiretroviral therapies and obtained positive outcomes regardless of the antiretroviral therapy taken, CD4 count and viral load. However, predictability of the long-term success of dental implants in HIV-positive patients has not been fully documented [7, 13–15].

The purpose of this study was to evaluate the success of dental implants placed in HIV-positive patients and provide insights to any trend of possible failures.

## Materials and methods

The protocol was approved by the Institutional Review Board (IRB) of Boston Medical Center (BMC). The Ryan White Foundation helps funding dental treatment for HIV patients at Henry M. Goldman School of Dental Medicine (GSDM) at Boston University [16, 17]. Information from that data pool was used to find potential participants. The criteria for selection were HIV-positive subjects with dental implant-related treatment. One hundred fifty subjects matched the criteria. The list was further narrowed to 70 potential subjects who had dental implant(s) placed at GSDM, who were 18 years of age or older, and had an existent and accessible dental record with all pertinent clinical and radiographic information on the dental implant(s) at the day of placement. The exclusion criteria were the following: pregnant women, patients diagnosed with HIV after implant placement, and missing records of radiographs. Seventy invitational letters of participation were sent and 18 out of the 70 patients contacted decided to return.

The follow-up time ranged from 7 months to 11.5 years post implant placement. This included records of implants placed between 2005 and 2017. The average follow-up was 3.6 years.

A standard questionnaire was filled out for every patient based on their dental records and interview done on the day of the appointment. Patients were divided into two groups: the Examination group that included the 18 patients which participated at the dental appointment and the Chart group which included 49 patients that did not attend but were eligible to be part of the research with complete information on their dental implant and health status including medications. Three subjects were deceased and therefore excluded.

Same data was collected for both groups except the Chart group did not include the follow-up radiographic examination or the patient’s own assessment regarding overall health, oral health, and dental hygiene habits. History of periodontal disease was evaluated through periodontal chartings and periodontal-related treatments rendered. Health-related topics such as systemic diseases (diabetes, myocardial infarction, and history of stroke) as well as social habits, such as smoking, were extracted from their records and during the interview at the research appointment for the Exam group.

A periapical radiograph was taken from the patients in the Exam group in order to evaluate the amount of bone loss compared to their initial radiograph taken at the day of implant placement.

## Results

Data from the survey was analyzed using SAS® (Statistical Analyses Software, version 9.3, Cary, NC).

*P* value showed no difference between the Exam and Chart groups. Altogether, the mean age was 58 years, 85% were male and 15% were female (Table 1). Forty-four percent of the patients responded excellent or very good, 44% good, and 11% poor regarding their overall health. For oral health, 44% of the patients responded excellent or very good, 44% good, and 11% poor. Seventy-seven percent brushed more than once a day and 23% brushed once a day. Eighty-two percent flossed more than once a day and 18% flossed once a day. Sixty percent of the patients in the Exam group were considered healthy (free of periodontal disease) upon clinical examination. Forty-six percent of the total (Exam and Chart groups) had a history of periodontal disease. Twenty percent of the patients in both groups were smokers, 20% were former smokers, and 60% have never smoked. Nine percent of the total had diabetes, 5% myocardial infarction, and 3% history of stroke.

**Table 1:** Demographics of participants

	Chart	Exam	Total	*P*
Total number of subjects	49	18	67	
Average age	58.2 (8.5)	59.1 (7.8)	58.4 (8.3)	0.6941
Gender				
Male	83.7%	88.9%	85.1%	0.7174
Female	16.3%	11.1%	14.9%	

Shows the number of participants, subdivided by charts screened before data collection and subjects who scheduled and participated in the exam and the total. Comparisons between the Chart group and the Exam group were done to determine if the results from the Exam group would reflect that of the Chart group. The groups were further divided by age and gender

A total of 142 implants were analyzed in both groups, 27 implants in the Exam group and 115 in the Chart group. Out of the 67 patients, 25 patients had one implant placed, 25 had two, and 17 had three or more dental implants. 80 percent of the total were partially edentulous.

Bone augmentation before or at implant placement was recorded as well. Eighty-seven percent of the patients had a bone augmentation procedure (socket graft, sinus lift, guided bone regeneration, bone graft at implant placement). Seventy-five percent were single-unit restorations and 25% were multi-unit. Forty-six percent were fixed screw-retained restorations, 45% were fixed cement-retained restorations, and 8.2% were removable restorations (overdentures).

One hundred percent of the patients took some kind of combination of ART. ART was classified by drug class also known as sub-category of medication: nucleoside reverse transcriptase (NRTI), non-nucleoside reverse transcriptase (NNRTI), protease inhibitors (PI), and integrase inhibitors (ISTI). The majority of the patients took one or more NRTI (94%). NRTI is considered the “backbone” of ART and tend to include a “third anchor” drug which may be a NNRTI, PI, or ISTI [18]. In this study, 34% took NNRTI, 33% PI, and 38% ISTI in combination with NRTI (Table 2).

**Table 2:** Subcategories of ART

Medication category	Total	Chart	Exam	*P*
ISTI	37.5%	31.8%	58.8%	0.0407
NRTI	93.8%	93.6%	94.1%	0.9437
NNRTI	33.8%	38.1%	17.6%	0.1136
PI	32.5%	34.9%	23.5%	0.3735

ART (antiretroviral treatment) drug classes in percentages. Combination of medication was separated to each drug class. Most if not all patients take NRTI alone or in combination with another ART drug

One hundred percent of the implants evaluated were assumed osseointegrated due to the lack of mobility. None of them showed signs of infection. None of the patients reported to have lost implants. Based on the classic criteria for success by which include the absence of pain, infection, neuropathies, paresthesia or violation of vital structures, implant immobility, no peri-implant radiolucency, negligible progressive bone loss (less than 0.2 mm annually after the first year of loading), and clinician and patient satisfaction [19, 20]. Only 1 out of the 18 implants evaluated did not succeed in the criteria, due to severe bone loss.

Radiographic examination evaluated the amount of crestal bone loss. Calibration and measurement of the radiographic images were done with ImageJ®. ImageJ® is a public domain, Java-based image processing program developed at the National Institutes of Health to calculate area and pixel value statistics [21]. To obtain accurate data, information of the diameter and length of the dental implant was used to calibrate the radiographic image. Once calibrated, bone loss was measured from the interface of the abutment and implant to the crestal bone on mesial and distal of the implant. The average bone loss on mesial was 0.6 mm and 0.3 mm on distal (Table 3). Only one implant had significant bone loss (more than 50%). Upon examination, the implant was in hyperocclusion but did not have bleeding upon probing, suppuration, or mobility.

**Table 3:** Bone loss measured in periapical radiograph

	Exam PA (mm)	*P*
Mesial bone loss (mm)	0.669	
Distal bone loss (mm)	0.351	
General bone loss (mean %)	5.2% (12.4%)	range 16.6–53.1%

Radiographic measurements of bone loss measured in 27 periapical radiographs of dental implants. Measured in millimeters (mm) using the diameter and length of the implant as reference and adjusted for distortion. Once calibrated, bone loss was measured on the radiograph taken in the research appointment. The time range from the radiograph taken after implant placement was between 6 months up to 5 years

## Discussion

The lack of complications shows that dental implants are a viable treatment for HIV-positive patients. Findings agree with similar reports found in the literature [12–15, 22]. Previous studies have shown low BMD is common in HIV-positive patients, and the frequency varies between 40 and 88% [23]. Low BMD may be related to comorbodities associated with osteoporosis such as increased age, smoking, low body mass index, or renal failure [10, 23]. Most of the patients in this study were over 50 years old and 11% were current smokers. Medical history was updated at the research appointment for the Examination group, and none of them mention renal failure or osteoporosis as systemic diseases.

Patients from the Exam group were highly compliant patients, and 88% considered their overall health as well as their oral health to be good or excellent. The Exam group maintained optimal hygiene and followed routine dental visits for maintenance. The absence of peri-implantitis demonstrates a high success rate in dental implants [24].

The main limitation of this study was the amount of data collected from the subjects and the number of subjects evaluated. Only 18 of the 67 potential subjects were willing to participate and data collection of 49 relied on the subject’s dental files. The study focused on the bone loss, mobility, and absence of infection as parameters of success. Although calibration of the radiographs was done, precise measurements of bone loss were not obtainable due to distortion from the angulation of the initial images. Ideally, periapical radiographs should be taken with a custom positional image device to obtain the same angulation as the image of the day of implant placement to be able to compare and evaluate progression of bone loss. This is a retrospective study, and variation between angulations was inevitable.

Crestal bone loss for a successful implant is less than 0.2 mm annually following the first year of function [19, 25, 26]. In the study, the average mesial bone loss was 0.7 mm and distal bone loss 0.4 mm over 3.6 years. Only one implant had enough bone loss to be considered failing; the mesial bone loss was 5.3 mm and 6 mm on distal, overall close to 50% loss. The crown was in hyperocclusion and the patient was referred for further evaluation and treatment.

The present study aimed to evaluate success of dental implants in HIV-positive patients. [19] None of the implants in the data showed risk of failure for evident reasons, due to HIV itself or the use of antiretrovirals. Dental implants are not contraindicated in HIV-positive patients[1, 6, 27].

None of the patients in the Chart group returned to the dental school with problems related to their dental implant including implant removal which implies the relative success. Implant success has been generally reported to be greater than 90% for fully and partially edentulous patients [28, 29]. The present study reported 94% success rate of the implants evaluated in the Exam group which were able to be fully evaluated with the implant success criteria. Other than the one failing implant in a single subject, the rest of the implants evaluated were considered successful. This study supports and agrees that dental implants maintained with good oral hygiene will be successful regardless of the patient’s medical status in this case HIV positive and the usage of ART.

Low BMD due to HIV itself or ART may lead to increased risk of bone fracture and osteoporosis [8, 10, 11]. Although bone quality was not evaluated, the results in crestal bone loss show indifference between dental implants in HIV-positive and the non-HIV patients. Although longer follow-ups are recommended to establish long-term success, the use of dental implants is a reasonable, well-tolerated and predictable treatment for HIV-positive patients taking ART [5, 30].

## Conclusion

Within the limitations of this study, the results imply that the placement of dental implants on HIV-positive patients may be safe and effective. We acknowledge further investigation is needed on the relationship of HIV, antiretroviral therapy, and dental implants. The findings in this study agree with the existing literature that modifiable factors such as hygiene may play a higher role.
